# Identification, Characterization, and Structure of Tm16 from* Trichuris muris*

**DOI:** 10.1155/2017/4342789

**Published:** 2017-08-14

**Authors:** Zhuyun Liu, Alan Kelleher, Shanii Tabb, Junfei Wei, Jeroen Pollet, Peter J. Hotez, Maria Elena Bottazzi, Bin Zhan, Oluwatoyin A. Asojo

**Affiliations:** ^1^National School of Tropical Medicine, Baylor College of Medicine and Texas Children's Hospital Center for Vaccine Development, Houston TX 77030, USA; ^2^Departments of Molecular Virology and Microbiology, Baylor College of Medicine, Houston, TX 77030, USA

## Abstract

Trichuriasis is a disease of poverty for which excretory and secretory (ES) products that induce the protective immunity are being investigated as candidate vaccines antigens. In this study, ES products of* T. muris* and immune sera were produced. The immune sera recognized more than 20 proteins on a 2D-gel of ES products of* T. muris* adult worms. Tm16 was one of the proteins identified by mass spectrometry. Tm16 shares 57% sequence identity with Ov16, an immunodominant diagnostic antigen from* Onchocerca volvulus*. Recombinant Tm16 with a carboxyl terminal hexahistidine was produced using* Pichia pastoris. *Polyclonal antibodies against rTm16 were generated by one-prime and two-boost immunization of three female Balb/c mice with 25 *μ*g of recombinant Tm16 emulsified with ISA720 adjuvant. These polyclonal antibodies confirmed that Tm16 is localized to the ES products and the soluble fraction of the adult worm. Additionally, the high-resolution crystal structure of Tm16 was solved by molecular replacement. Tm16 belongs to the phosphatidylethanolamine-binding-like protein (PEBP1) family and this is the first structure of a PEBP1 from a parasite.

## 1. Introduction

*Trichuris trichiura*, one of the three most common soil-transmitted nematodes, causes trichuriasis in more than 450 million people and an estimated 544,000 disability adjusted life years globally according to the Global Burden of Disease Study 2015 [1]. Trichuriasis remains a problem in the USA, with 13% of school children in Clay County, Kentucky, infested with* Trichuris trichiura *[2–4]. Trichuriasis is also a health concern for the poor in rural areas of the gulf coast, Appalachia, tribal lands, and inner cities, and for refugee communities, prisoners, mental health patients, migrant workers, and children in all parts of the country who are allowed to play in soil or sand that could be contaminated [3, 4]. The current approaches for soil-transmitted helminth infections such as trichuriasis include mass drug administration, but the major drugs (mebendazole, albendazole) used to treat trichuriasis have a low (28–36%) cure rate [5] and do not completely break the cycle of reinfection [6]. This observation explains why the global prevalence of human whipworm infection has decreased only 2.1% over the last decade [1], such that there is a vital need for alternative therapies that ameliorate the health of infected people in order to alleviate the global health and economic and social burdens of NTDs. Currently, trichuriasis is diagnosed using fecal egg counts and there is a need to develop additional diagnostic methods.

One possible approach is to identify diagnostics or vaccine antigens for* T. trichiura *using the mouse equivalent,* Trichuris muris*, as a model. Like* T. trichiura, T. muris is *a whipworm with a long and narrow head embedding in the epithelial layer of large intestine of the host. There is precedence for characterizing ES products as candidate vaccine antigens for parasites. ES products are known to suppress host immune response and facilitate parasitism in the hostile environment of the host [7, 8]. Mice immunized with some ES products produced almost sterile protective immunity against challenge* of T. muris* infective eggs [7, 8]. Our vaccine discovery efforts include the identification and characterization of ES products that induce the protective immunity as vaccine candidates. We present here the identification, production, and crystal structure of Tm16, a whipworm ES protein. Tm16 shares 57% amino acid identity with Ov16, an immunodominant diagnostic antigen of* Onchocerca volvulus. *Ov16 was identified from sera of West African Onchocerciasis patients and determined to be a selective antigen that is recognized only by sera from people infected with* Onchocerca volvulus*, but not people infected with other filarial parasites [9].

Based on its amino acid sequence, Tm16 belongs to the PEBP and DOCK1 superfamily. PEBP is highly conserved in organisms including bacteria, yeast, nematodes, plants, drosophila, and mammals [10] with functions involved in the control of several signaling pathways by interacting with other cellular components including the inhibition of the MAP kinase pathway [10], the NF-*κ*B pathway [11], regulation of the action of heterotrimeric G proteins [12], and serine protease inhibition [13]. PEBP also acts as a kinase regulator controlling the morphological switch between shoot growth and flower structures [14]. DOCK1 (also called DOCK180) coordinates with ELMO1 to regulate the small GTPase Rac, thereby influencing several biological processes, including phagocytosis, cell migration, and signal pathway. Dock1 in* Caenorhabditis elegans* plays a critical role in Rac-dependent cell migration that is essential throughout the embryonic and adult life of the nematode [15]. DOCK180 is an effector molecule which transduces signals from tyrosine kinases through the CRK adaptor protein [16]. Farnesylated DOCK180 can drive cell spreading, implying that it is involved in the regulation of cell movement by tyrosine kinases. Some research suggested DOCK1-like protein was involved in the cytoskeletal reorganization required for an engulfing cell to extend its surface around a dying cell during phagocytosis [17].

## 2. Materials and Methods 

### 2.1. Production of Excretory and Secretory (ES) Products of* T. muris* and Immune Sera

ES products were produced using established protocols [18–25]. ES products were obtained from the overnight culture of* T. muris* adult worms isolated from laboratory maintained STAT6/KO mice. The concentrated* T. muris* ES products were used to immunize AKR mice, generate antisera, and test vaccine efficacy against* T. muris* infection. Each mouse was subcutaneously immunized with 100 *μ*g ES products formulated with ISA720 (Seppic, France) three times with 2-week interval. The antisera (mouse anti-ES sera) were obtained from immunized mice 10 days after the last immunization and the immunized mice were subsequently challenged with 300* T. muris* embryonated eggs.

### 2.2. Electrophoresis and Immunoblotting of ES Products

The anti-ES sera were collected from mice immunized with* T. muris* ES products. The mouse anti-ES sera were used to identify ES products separated on a 2D gel as previously described [26]. Briefly, 100 *μ*g/600 *μ*g of* T. muris* ES products were separated on two 2D gels. The gel loaded with 100 *μ*g of* T. muris* ES products was transferred on a PVDF membrane, while that loaded with 600 *μ*g of* T. muris* ES products was stained with Coomassie brilliant blue. The spots were recognized by Western blotting using mouse anti-ES immune sera as primary antibody and HRP-conjugated anti-mouse IgG (Invitrogen, US, 1 : 5,000) as secondary antibody. Spots were visualized by ECL chemiluminescence (Thermo Scientific, US). There were more than 20 protein spots recognized by the immune sera. Ten of the corresponding proteins of the recognized spots on the Coomassie-stained gel were identified by matching with immunoblot image and excised.

### 2.3. Protein Identification and Liquid Chromatography Tandem Mass Spectrometry (LC-MS/MS)

Ten spots were excised from the 2D-PAGE gel of the ES products and sent to Keck Biotechnology Resource Laboratory at Yale University for protein identification using liquid chromatography with tandem mass spectrometry (LC-MS/MS). Once received at Keck Biotechnology Center, spots were washed with 50% acetonitrile for 10 min with rocking and then washed with 50% acetonitrile/50 mM NH_4_HCO_3_. After a final wash with 50% acetonitrile/10 mM NH_4_HCO_3_, the gel spots were dried by speed vacuum. Each spot was resuspended in 35 *μ*l of 10 mM NH_4_HCO_3_, containing 0.25 *μ*g of digestion grade trypsin (Promega, V5111), and incubated at 37°C for 14 hours.

LC-MS/MS analysis was performed on a Thermo Scientific Orbitrap Elite equipped with a Waters nanoAcquity UPLC system utilizing a binary solvent system (Buffer A: 100% water, 0.1% formic acid; Buffer B: 100% acetonitrile, 0.1% formic acid). Trapping was performed at 5 *μ*l/min, 97% Buffer A for 3 min using a Waters Symmetry® C18 180 *μ*m × 20 mm trap column. Peptides were separated using an ACQUITY UPLC PST (BEH) C18 nanoACQUITY Column 1.7 *μ*m, 75 *μ*m × 250 mm (37°C) and eluted at 300 nl/min with the following gradient: 3% buffer B at initial conditions; 10% B at 1 minute; 35% B at 38 minutes; 90% B at 43 minutes; 90% B at 48 min; return to initial conditions at 50 minutes. MS was acquired in the Orbitrap in profile mode over the 300–1,800 *m*/*z* range using 1 microscan, 30,000 resolution, AGC target of 1E6, and a full max ion time of 50 ms. Up to 15 MS/MS were collected per MS scan on species reaching an intensity threshold of 3,000 (charge states one and above). Data dependent MS/MS were acquired in centroid mode in the ion trap using 1 microscan, 15,000 resolution, AGC target of 2E4, full max IT of 100 ms, 2.0 *m*/*z* isolation window, and CID fragmentation with a normalized collision energy of 35. Dynamic exclusion was enabled with a repeat count of 1, repeat duration of 30 s, exclusion list size of 500, and exclusion duration of 60 s.

Data were searched in-house using the Mascot algorithm (Matrix Science; version 2.5.1) for uninterpreted MS/MS spectra after using the Mascot Distiller program to generate peak lists. The data was searched against an NCBInr database. Search parameters used were trypsin digestion with up to 2 missed cleavages; peptide mass tolerance of 10 ppm; MS/MS fragment tolerance of +0.5 Da; and variable modifications of Met oxidation and propionamide adduct to Cys. Normal and decoy database searches were searched to determine the false discovery rate, with the confidence level set to 95% (*p* < 0.05).

### 2.4. Production of Recombinant Tm16 Protein

DNA encoding the full length Tm16 was amplified from the total first-strand cDNA of adult* T. muris* and cloned into the* Pichia pastoris* expression vector pPICZ*α*A (Invitrogen, USA), using the EcoRI and NotI restriction sites to add a C-terminal hexahistidine tag. The correct open reading frame (ORF) was confirmed by sequencing using the vector flanking primers corresponding to the regions encoding the *α*-factor and 3′AOX1 genes. The recombinant plasmids were linearized following digestion with SacI and transformed into* P. pastoris* X33 strain by electroporation. A single colony was selected from zeocin-resistant YPD plates and recombinant Tm16 protein (rTm16) expression was induced in media containing 0.5% methanol for 72 hours. The culture supernatant containing the secreted rTm16 was isolated by centrifugation and filtered with 0.22 *μ*m PES filter top. The rTm16 was purified by Ni immobilized metal affinity chromatography (IMAC) and eluting with an imidazole gradient in the same buffer. The purified protein was dialyzed against TBS pH 7.5 to remove imidazole, concentrated to 1.6 mg/ml, and stored at −80°C.

### 2.5. Crystallization, Data Collection, and Structure Determination

The rTm16 was crystallized as flat plates at 289 K using vapor diffusion. Sitting drop contained 1.5 *μ*L of 22 mg/ml rTm16 in 5 mM Bis(2-hydroxyethyl)aminotris(hydroxymethyl) methane pH 6.5 and 1.5 *μ*L of the precipitant solution (0.1 M HEPES pH 7.5, 10% (v/v) isopropanol, 20% (w/v) PEG 4000), while the reservoir contained 300 *μ*L of precipitant solution. Crystals of dimension 0.2 mm × 0.05 mm × 0.5 mm (Figure S.1 in Supplementary Material, available online at https://doi.org/10.1155/2017/4342789) grew within 48 hours and the largest of these crystals diffracted to ~1.7 Å on the home source.

Crystals were flash-cooled directly in a stream of N_2_ gas at 113 K prior to collecting diffraction data at the Baylor College of Medicine core facility (Rigaku HTC detector, Rigaku FR-E+ SuperBright microfocus rotating anode generator, with VariMax HF optics) using the Crystal Clear (d^*∗*^trek) package [27]. Data was integrated using MosFLM and scaled with SCALA [28]. Data collection and processing statistics are summarized in Table 1.

Tm16 structure was solved by molecular replacement (MR) using PHASER [29, 30] with the crystal structure of human phosphatidylethanolamine-binding protein pdb code 1BEH [31] stripped of all ligands and Waters as search model. The deposited model was obtained by model building with Coot [32] and structure refinement with PHENIX [33]. Structural figures were generated using PyMOL [34]. Structure solution and refinement statistics are summarized in Table 1. Quality of the electron density maps is illustrated in Figure S.2.

### 2.6. Size-Exclusion Chromatography and Multiangle Light Scattering (SECMALS)

The rTm16 was concentrated and buffer exchanged to 15 mg/ml in PBS using a 10 kDA cut-off filter (Amicon Ultra-0.5 mL Centrifugal Filters). 25 *μ*L of rTm16 was injected onto a Phenomenex Yarra 3 *μ*m SEC-2000 column (Phenomenex, Torrance, CA) at flow-rate of 0.5 ml/min using an Agilent 1260 Infinity series HPLC. The mobile phase was PBS buffer at pH 7.4. The elution was detected with a UV detector (Agilent), a miniDAWN triple-angle light scattering detector (Wyatt Technology), and an Optilab rEX differential refractometer (Wyatt Technology) connected in series. The protein concentration was monitored across the peak using the protein extinction coefficient at 280 nm. The isotropic scatterer for detector normalization was bovine serum albumin. Molecular mass was calculated from the light scattering and interferometric refractometer data using ASTRA 6.1 software.

### 2.7. Generation of Mouse Antiserum and Western Blotting

For generating polyclonal antibodies against Tm16, three female Balb/c mice were subcutaneously immunized with 25 *μ*g of recombinant Tm16 (rTm16) emulsified with ISA720 adjuvant (Seppic, France), followed by two boosts at 3-week intervals. Fourteen days after the last boost, the mice were euthanized, their blood was collected, and sera was isolated and pooled. The resulting mouse anti-rTm16 sera was aliquoted and stored at −20°C. The localization of native Tm16 in* T. muris* adult ES products was determined by Western blotting using mouse anti-rTm16 sera. Total 5.0–10.0 *μ*g of* T. muris* adult ES was separated on a precast 4–20% gradient SDS polyacrylamide gel (Invitrogen) transferred onto a PVDF membrane (Millipore). The native Tm16 was probed with a 1 : 4000 dilution of mouse anti-Tm16 sera and visualized with HRP-conjugated anti-mouse IgG (Invitrogen, US, 1 : 5,000) and ECL chemiluminescence (Thermo scientific, US). 50 ng of rTm16 and rTm14-3-3, another recombinant* T. muris* protein, were used as positive and negative control, respectively.

### 2.8. Phylogenetic Tree Generation

The phylogenetic tree was generated using one click analysis mode online at http://www.phylogeny.fr. MUSCLE 3.8.31 was used for multiple sequence alignment while PhyML 3.1 for phylogeny and TreeDyn 198.3 was used for tree rendering.

## 3. Results

### 3.1. Identification of Tm16

**Figure 1:**
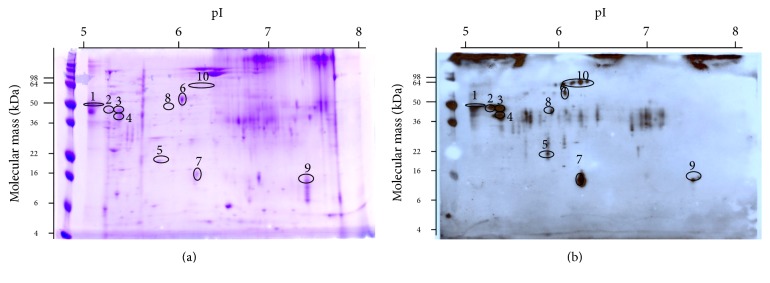
Identification of Tm16 by 2D gel electrophoresis on 8–16% Tris-HCl criterion gel. The first dimension was isoelectric focusing while the second dimension was SDS-PAGE. Ten independent protein spots were excised and sent for identification. Tm16 identified in spots 7 and 9. (a) Gel was stained with Coomassie brilliant blue. (b) Western blot of corresponding gel primary antibody was mouse ES immune sera and secondary antibody was anti-mouse IgG HRP.

The* T. muris* excreted products were separated on 2D gel and visualized with Coomassie staining (Figure 1(a)) or probed with mouse anti-ES immune sera (Figure 1(b)). The Coomassie-stained gel and immunoblot gel were aligned and matched. Ten of the overlapped spots were excised for protein identification by mass spectrometry (MS). Through a BLAST search against the GenBank database, a 187 amino acid protein sharing 57% amino acid identity with Ov16, an immunodominant antigen of* Onchocerca volvulus *[9], and 86% identity with Tt16 from human* T. trichiura* (CDW60800.1) was identified and named Tm16. The major proteins identified by MS are detailed in Table 2.

Tm16 was identified in spots 9 and 7 which are written in bold in Table 2. The confidence scores for the identification of each peptide from the Tm16 protein provided by MASCOT are provided in Table 3 as evidence for the identification of the protein in each separated spot.

**Figure 2:**
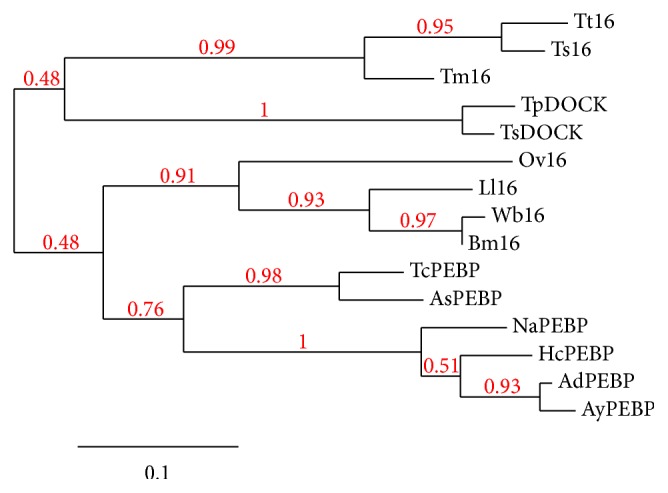
Phylogenetic tree comparison of Tm16 and homologues from other nematodes, showing branch support values in red. Tt16 (*Trichuris trichiura*, GenBank: CDW60800.1); Ts16 (*T. suis*: KHJ42858.1); TpDOCK (*Trichinella pseudospiralis*: KRX98670); Ll16 (*Loa loa*: EJD73732.1); AdPEBP (*Ancylostoma duodenale*: KIH55180.1); TcPEBP (*Toxocara canis*: KHN87196.1); TsDOCK (*Trichinella spiralis*: KRY40094.1); HcPEBP (*Haemonchus contortus*: CDJ94417.1); AsPEBP (*Ascaris suum*: ERG86178.1); AyPEBP (*Ancylostoma ceylanicum*: EYB84014.1); Wb16 (*Wuchereria bancrofti*: EJW88954.1); NaPEBP (*Necator americanus*: XP_013301336.1); Bm16 (*Brugia malayi*: CRZ25715.1); and Ov16 (*Onchocerca volvulus*: P31729.2).

Phylogenetic tree comparison of Tm16 reveals that it belongs to the same branch as Ov16 (Figure 2). Tm16 belongs to the phosphatidylethanolamine-binding-like protein (PEBP) and dedicator of cytokinesis protein 1 (DOCK1) superfamily (Figure 2). The PEBP superfamily is highly conserved in organisms including bacteria, yeast, nematodes, plants, drosophila, and mammals [10]. PEBP are involved in the control of several signaling pathways by interacting with other cellular components including the inhibition of the MAP kinase pathway [10], the NF-*κ*B pathway [11], regulation of the action of heterotrimeric G proteins [12], and serine protease inhibition [13] and acting as a kinase regulator controlling the morphological switch between shoot growth and flower structures [14].

### 3.2. Production of rTm16 and Native Tm16 Localization

**Figure 3:**
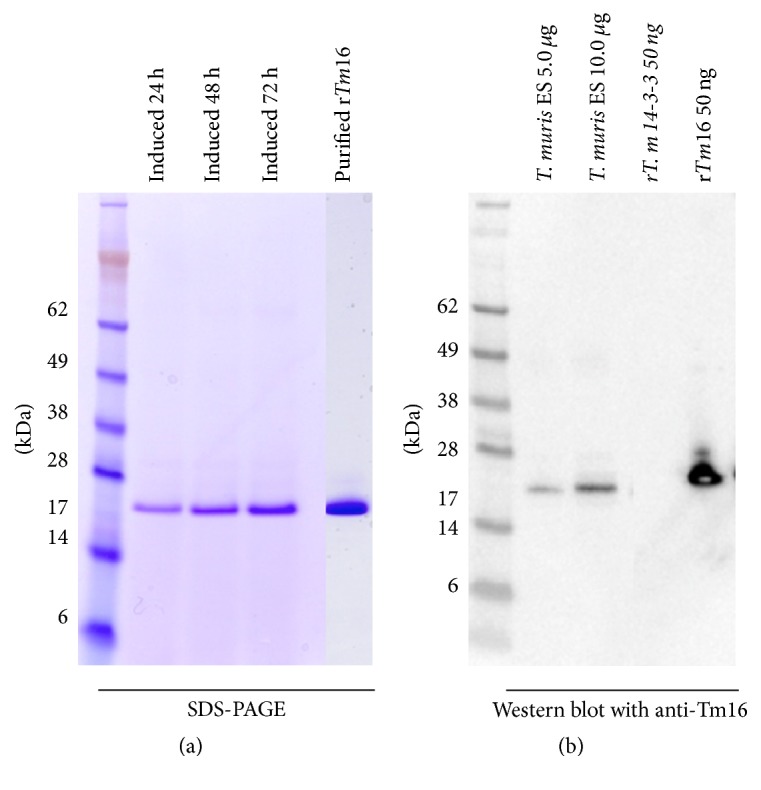
Production and localization of Tm16. (a) SDS-PAGE of ~2 *μ*g rTm16 expressed in* P. pastoris* X-33 after being induced with 5% methanol for 24–72 hours and IMAC purification. (b) Western blot showing the native Tm16 is in the* T. muris* ES products; rTm16 is slightly larger than the protein in the ES because it has a hexahistidine tag. The rTm14-3-3 (50 ng) was used as a negative control.

rTm16 is highly expressed as soluble protein in yeast Pichia pastoris X-33 by methanol induction and could be purified to ~99% purity by IMAC (Figure 3(a)). Antiserum generated against rTm16 (mouse anti-rTm16) was specific enough to determine the localization of native Tm16 in* T. muris* worms by Western blotting and demonstrated that native Tm16 is localized in the* T. muris* adult ES products (Figure 3(b)). Mouse anti-rTm16 was also specific for Tm16 and does not recognize another recombinant hexahistidine tagged* T. muris* antigen Tm-14-3-3 (Figure 3(b)). The recombinant Tm16 appeared as ~1 kDa higher than native Tm16 since rTm16 contains a hexahistidine tag expressed at C-terminus.

### 3.3. Structure of Tm16

**Figure 4:**
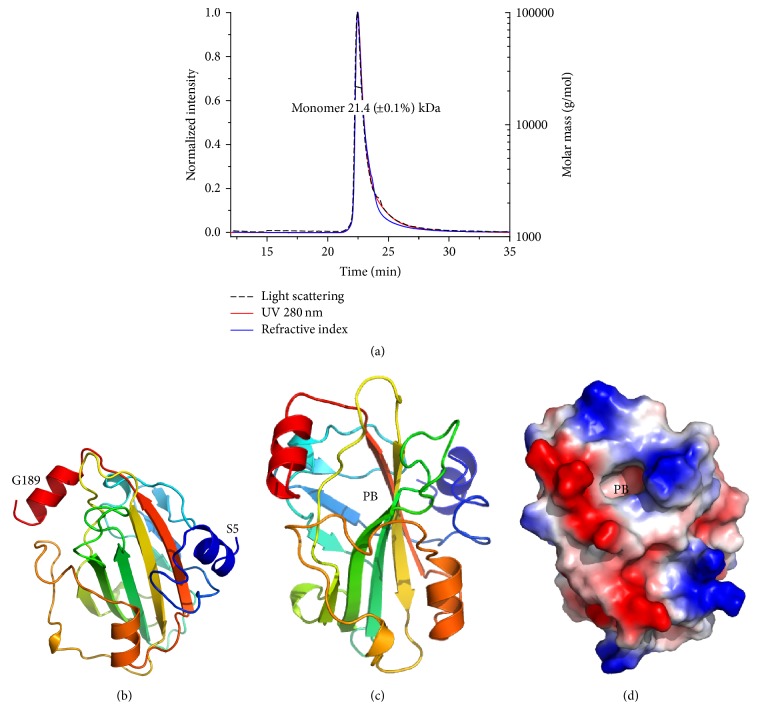
(a) Tm16 is an ~21 kDa monomer in solution according to size-exclusion chromatography and multiangle light scattering analysis. (b) Ribbon diagram of Tm16 monomer colored in rainbow from blue (N-ter) to red (C-ter). The central putative binding cavity (PB) is visible in ribbon diagram (c) and electrostatic surface plot (d).

The structure of Tm16 solved by molecular replacement has a monomer in the asymmetric unit. Like the crystal structure, rTm16 is monomeric in solution and the solution molecular mass determined by SECMALS is ~21.4 kDa (Figure 4(a)). The atomic coordinate and structure factors have been deposited in the protein data bank under accession number 5TVD. Tm16 has the typical topology of a phosphatidylethanolamine-binding-like protein (PEBP), having four helices and nine beta strands that include the central six-strand beta sheet of the PEBP fold (Figure 4(b)). A large central cavity corresponding to the putative PEBP ligand-binding pocket is located at the end of the central beta sheet (Figures 4(c) and 4(d)).

## 4. Discussion

**Figure 5:**
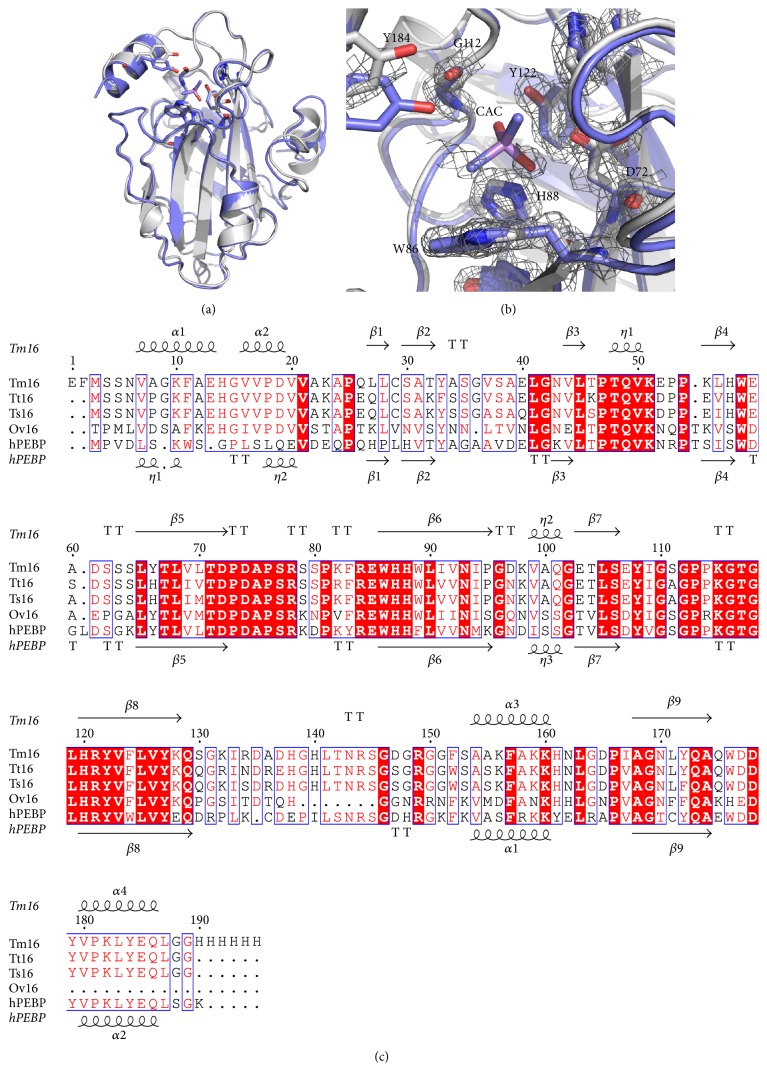
(a) Alignment of hPEBP (blue) Tm16 (gray). The putative active site residues are shown in stick. (b) The putative binding cavity of Tm16 is open enough to accommodate cacodylate (CAC), a ligand found bound in hPEBP. The electron density map contoured at 1.6*σ* (gray mesh) shows that there are no ligands bound in the cavity or the Tm16 structure. (c) Structural and primary sequence alignment of Tm16, Tt16, Ts16, Ov16, and hPEBP. The secondary structure elements shown are alpha helices (*α*), 3_10_-helices (*η*), beta strands (*β*), and beta turns (TT). Identical residues are shown in white on red background and conserved residues in red. Figure generated using Espript [35, 36].

The structures most similar to Tm16 were identified by 3D structural alignment using PDBeFold's structure similarity option (http://www.ebi.ac.uk/msd-srv/ssm/) and the most similar structure was the human phosphatidylethanolamine-binding-like protein (hPEBP) [31] followed by homologues from other mammals. The main chain atoms of the Tm16 monomers align with hPEBP with an rmsd of 0.456 Å for all main chain atoms (Figure 5(a)). Additionally, the putative binding cavity of Tm16 also aligns well with that of hPEBP with sufficient space to accommodate ligands (Figure 5(b)). The structure of Tm16 can serve as a suitable model to predict the structure of other parasite phosphatidylethanolamine-binding-like proteins based on predicted topology and sequence conservation (Figure 5(c)).

The crystal structure of Tm16 is the first structure of a parasite PEBP and reveals a prototypical phosphatidylethanolamine-binding-like topology with a large binding cavity capable of accommodating various ligands and suggestive of the ability to bind with macromolecules related to the signal pathway and transduction or cell migration and regulation (Figures 4 and 5). Since Tm16 shares extensive structural similarity with hPEBP, it may have similar functions.

Given that Tm16 is one of the* T. muris* secreted proteins that induce protective immunity in immunized mice, it can be investigated as a putative vaccine candidate for preventing* Trichuris *infection. The high yield expression of Tm16 as a soluble recombinant protein in a scalable reproducible* P. pastoris *system is the first step towards developing it as a vaccine candidate for vaccine trial using our* T. muris* mouse model. Due to the similarity of Tm16 to Ov16 it may instead be more suitable as a diagnostic antigen. More studies are required to determine if Tm16 functions as a biofunctional PEBP/DOCK1 regulatory molecule and what effects these putative functions have on future applications of Tm16.

## 5. Conclusion

Tm16 was identified as part of antigen discovery efforts, and methods were developed for the production and purification of Tm16. Its similarity to Ov16 makes it a promising diagnostic antigen. The recombinant protein produced was monodisperse and pure and was used for structure determination. Tm16 is the first structure of a parasite PEBP and reveals significant structural similarity to mammalian PEBP. The roles of Tm16 in the survival of parasite in host, the pathobiology of human trichuriasis, and host-parasite interactions based on its putative functions in ligand binding and cell signaling are topics for future investigation.

## Supplementary Material

Figure S.1: Sample crystals of Tm16.
Figure S.2: Fit of Tm16 in 2Fo-Fc Electron density maps contoured at 1.6sigma.

## Figures and Tables

**Table 1:** Data collection and refinement statistics.

	Tm16 (5TVD)
Wavelength	0.15418 nm
Space group	C 1 2 1
*a*, *b*, *c* (Å)	*a* = 85.97 Å, *b* = 31.7 Å, *c* = 63.75 Å
*α*, *β*, *γ* (°)	*α* = *γ* = 90.00° *β* = 97.3°
Mosaicity (°)	0.8
Resolution range (Å)	63.23–1.73 (1.83–1.73)
Total number of reflections	31537 (2915)
Number of unique reflections	17974 (1741)
Completeness (%)	99.25 (98.20)
Redundancy	1.8 (1.7)
〈*I*/*σ*(*I*)〉	10.05 (3.58)
*R*_r. i. m._	0.04017 (0.1777)^†^
Overall *B* factor from Wilson plot (Å^2^)	16.31
CC (free)	0.935 (0.637)
Number of nonhydrogen atoms	1654
Macromolecules	1418
Ligands	8
Solvent	228
Protein residues	185
RMS (bonds)	0.009
RMS (angles)	1.02
Ramachandran favored (%)	98
Ramachandran allowed (%)	2.2
Ramachandran outliers (%)	0
Rotamer outliers (%)	2
Clash score	1.42
Average *B*-factor	19.82
Macromolecules	18.25
Ligands	9.55
Solvent	29.94

Statistics for the highest-resolution shell are shown in parentheses. ^†^Estimated *R*_r. i. m._ = *R*merge[*N*/(*N* − 1)]1/2, where *N* is data multiplicity.

**Table 2:** Proteins identified by mass spectrometry (Tm16 is written in bold).

Protein orthologue name	Protein ID	Score	Expectation	MW	% coverage	Gel spot #
Heat shock protein 70 [*T. trichiura*]	gi | 669222654	1503	0	130217	23.9	10
78 kDa glucose regulated protein [*T. trichiura*]	gi | 669221950	915	2.30*E* − 84	72739	30.2	10
Intermediate filament protein ifa 1 [*T. trichiura*]	gi | 669224300	748	1.20*E* − 67	70668	13.9	5
T complex protein 1 subunit beta [*T. trichiura*]	gi | 669219796	587	1.20*E* − 51	58952	24.4	6
Hypothetical protein M513_02789 [T. suis]	gi | 669312874	551	5.00*E* − 48	69632	10.7	5
Calreticulin [*T. trichiura*]	gi | 669220004	491	5.80*E* − 42	50744	21.5	2, 3, 4, 6, 8
Hypothetical protein M513_03661 [*T. suis*]	gi | 669311855	451	5.00*E* − 38	72481	17.4	10
Enolase [*T. trichiura*]	gi | 669226327	422	4.20*E* − 35	49481	22.2	1, 5
Eukaryotic translation elongation factor 1A [*T. trichiura]*	gi | 669225571	394	2.80*E* − 32	51053	15.7	1
Putative heat shock protein [*T. trichiura*]	gi | 669221150	391	5.00*E* − 32	22276	35.4	5
14-3-3 protein [*T. trichiura*]	gi | 669225856	366	1.70*E* − 29	33188	17.9	5
Peptidyl-prolyl cis-trans isomerase [*T. trichiura*]	gi | 669222452	325	2.10*E* − 25	22561	32.2	7, 9
Hypothetical protein M513_06612 [*T. trichiura*]	gi | 669309085	308	1.00*E* − 23	54982	10.2	6
Phosphoenolpyruvate carboxykinase GTP [*T. trichiura*]	gi | 669222197	273	3.20*E* − 20	70928	11.3	10
Hypothetical protein TTRE_0000417601 [*T. trichiura]*	gi | 669222794	257	1.30*E* − 18	25963	14.9	5, 7
Glyceraldehyde 3 phosphate dehydrogenase [*T. trichiura*]	gi | 669218531	254	2.70*E* − 18	37512	16	10, 8
Peptidyl prolyl cis trans isomerase FKBP4 [*T. trichiura*]	gi | 669223960	234	2.80*E* − 16	48563	10.4	8
Hypothetical protein M513_00518 [*T. trichiura*]	gi | 669315377	168	9.90*E* − 10	45230	10.4	8
Major sperm protein [*T. trichiura*]	gi | 669225999	156	1.60*E* − 08	22126	14.5	5
**OV 16 antigen [*T. trichiura*]**	**g** **i** | 669215881	**155**	**0.00000002**	**20422**	**14.4**	**9, 7**
Mediator of RNA polymerase II transcription subunit 22 [*T. trichiura*]	gi | 669225844	138	0.0000012	15475	21.6	10
Peroxiredoxin-2 [*T. trichiura*]	gi | 669217645	104	0.0027	22076	10.6	7

**Table 3:** Mascot confidence scores for peptides in spots 7 and 9.

Spot	Peptide	Score	Expectation	Start	End	*M/Z*	Ion mass	Ion mass (calc)	Delta
7	K.FAEHGVVPDVVAK.A	63.85	0.0094	9	21	684.3678	1366.7211	1366.7245	−0.0034
7	R.YVFLVYK.Q	41.55	2.8	120	126	466.2675	930.5205	930.5215	−0.001

9	K.FAEHGVVPDVVAK.A	77.27	0.00049	9	21	684.3686	1366.7227	1366.7245	−0.0018
9	R.YVFLVYK.Q	44.48	1.4	120	126	466.2683	930.5221	930.5215	0.0006
9	K.LYEQLGG.-	33.69	8.3	181	187	779.3921	778.3848	778.3861	−0.0013
