# Supplementary figures and images for: Identification, Characterization, and Structure of Tm16 from Trichuris muris
Source: J Parasitol Res. 2017 Aug 14;2017:4342789. doi: 10.1155/2017/4342789 (PMC5573103; doi:10.1155/2017/4342789)

## Slide 1
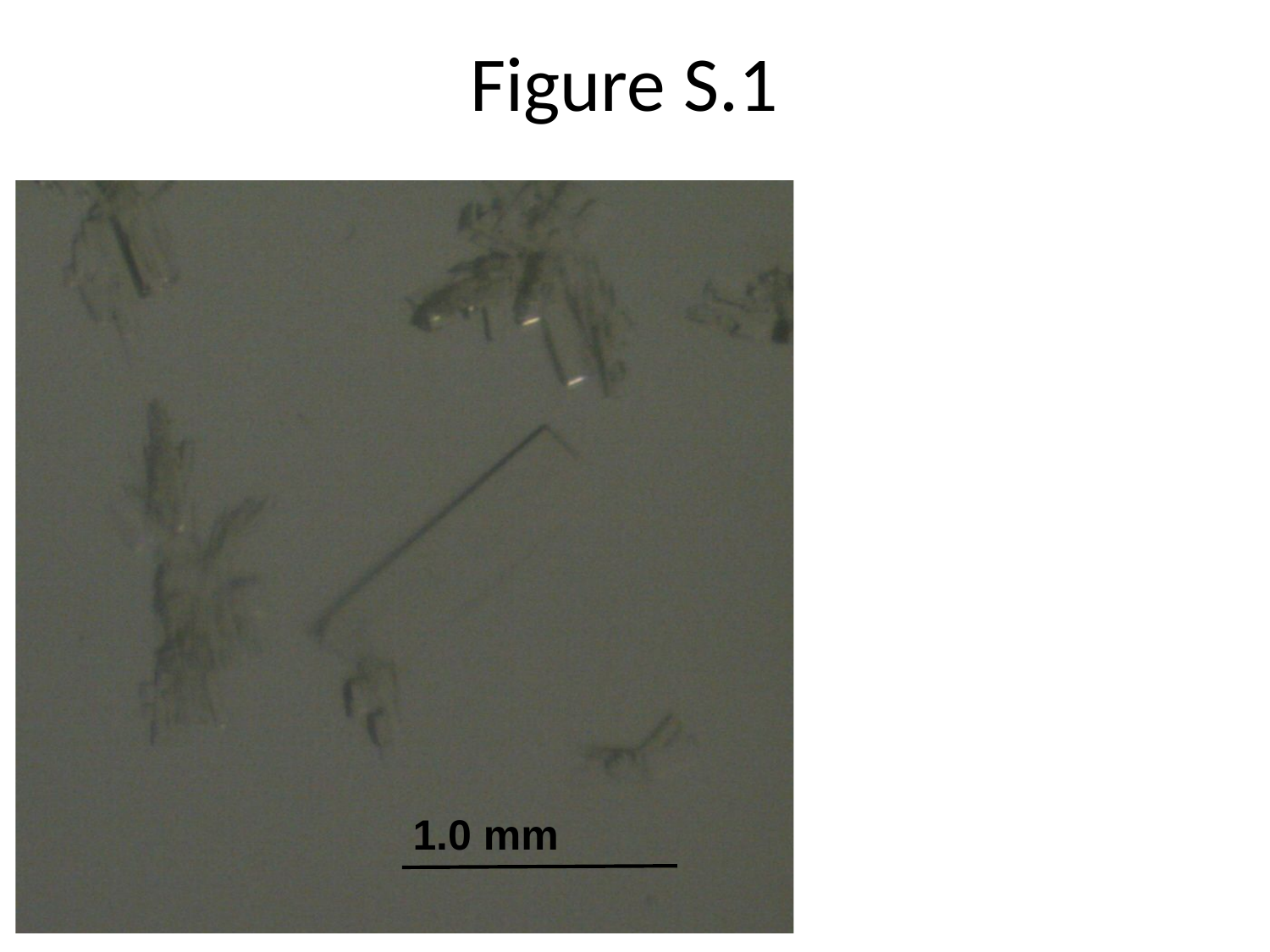

# Figure S.1
1.0 mm

## Slide 2
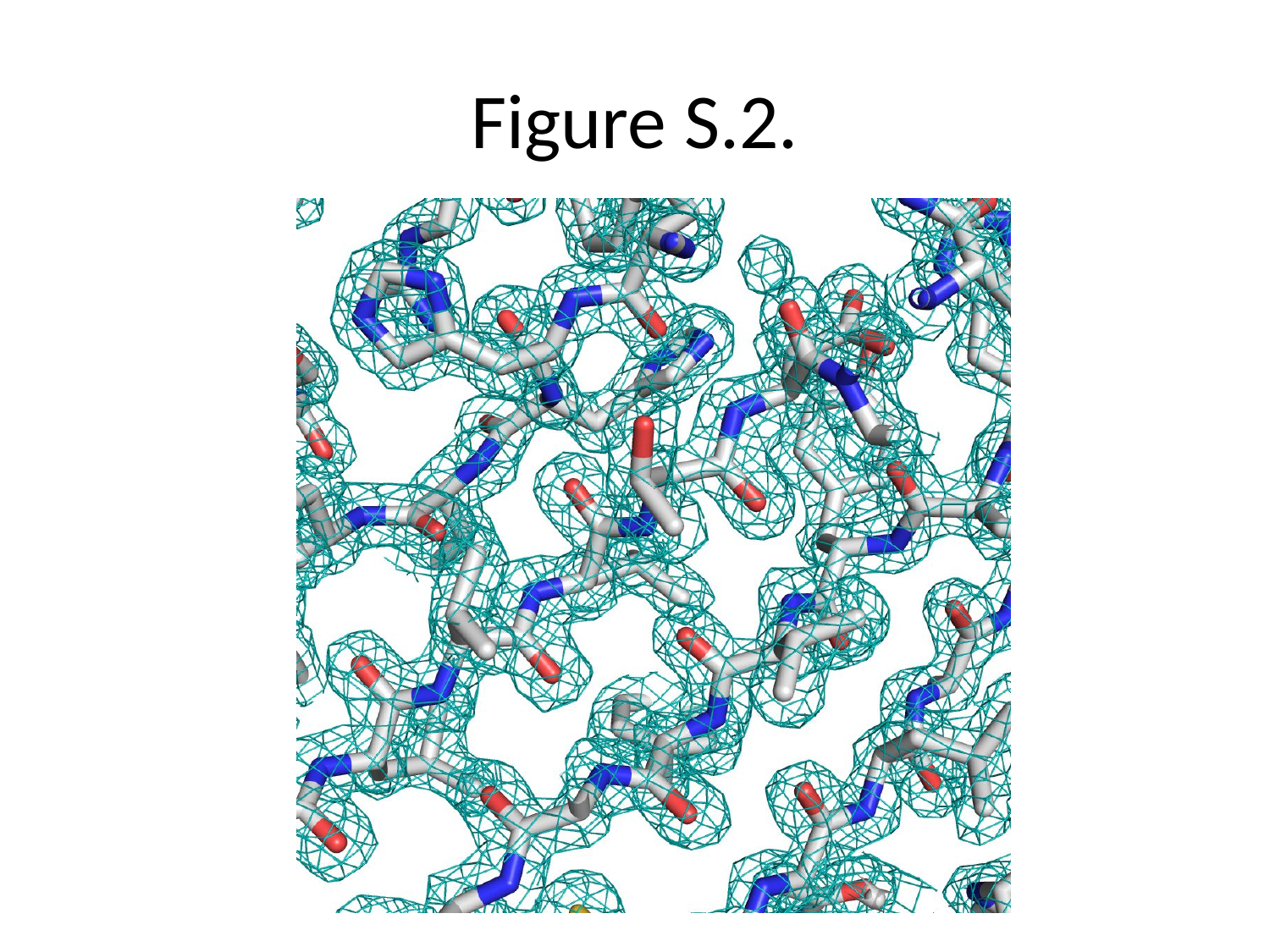

# Figure S.2.

Supplement: Supplementary file 1 — Figure S.1: Sample crystals of Tm16. Figure S.2: Fit of Tm16 in 2Fo-Fc Electron density maps contoured at 1.6sigma. [file 4342789.f1.pptx]
